# Respiratory Symptoms and Lung Function in Never-Smoking Male Workers Exposed To Hardwood Dust

**DOI:** 10.3889/oamjms.2015.086

**Published:** 2015-07-16

**Authors:** Dragana Bislimovska, Sunchica Petrovska, Jordan Minov

**Affiliations:** 1 *Department of Medical and Experimental Physiology with Anthropology, Medical Faculty, Ss Cyril and Methodius University of Skopje, Skopje, Republic of Macedonia*; 2 *Institute of Occupational Health of Republic of Macedonia, WHO Collaborating Center, Skopje, Republic of Macedonia*

**Keywords:** respiratory symptoms, questionnaire, spirometry, wood dust, workplace exposure

## Abstract

**BACKGROUND:**

Results from many studies suggest that workplace exposure to organic dust may lead to adverse respiratory effects in exposed workers.

**AIM:**

In order to assess the respiratory effects of the workplace exposure to hardwood dust we performed a cross-sectional study of never-smoking male workers employed in parquet manufacture and never-smoking male office workers as a control.

**MATERIAL AND METHODS:**

We performed a cross-sectional study including 37 never-smoking male workers employed in parquet manufacture and an equal number of never-smoking male office workers studied as a control. Evaluation of examined subjects included completion of a questionnaire for respiratory symptoms in the last 12 months and baseline spirometry performed according to the actual recommendations.

**RESULTS:**

We found a higher prevalence of respiratory symptoms in parquet manufacturers than in office workers with significant difference for cough and phlegm. Majority of the respiratory symptoms in the parquet manufacturers were work-related. The mean values of all spirometric parameters with exception of forced ventilatory capacity (FVC) were significantly lower in the parquet manufacturers as compared to their mean values in the office workers. We found close relationship between both the prevalence of respiratory symptoms and the reduction of spirometric parameters in the parquet manufacturers and the duration of the workplace exposure to wood dust.

**CONCLUSION:**

Our data suggest that workplace exposure to hardwood dust may lead to adverse respiratory effects indicating the need of adequate preventive measures in order to protect the respiratory health of exposed workers.

## Introduction

Results from a number of studies indicated that the exposure to organic dusts, such as cotton, flax, wood, paper, grain and other agricultural dusts, may lead to respiratory impairment and development of chronic lung disease, independently of any effect due to smoking [1-5].

Wood dust is created when machines are used to cut or shape wood materials. Industries with a risk of wood dust exposure include sawmills, dimension mills, secondary wood products manufacture (parquet, doors, windows, etc.), furniture manufacture, cabinet makers, and carpenters. During their working activities woodworkers may be exposed to different exposure types, i.e. hardwood or softwood dust, dust from natural wood or wood-based composites, pure wood dust or wood dust containing adhesives, paints and other chemicals, etc [6, 7]. Woodworking at the global level is dominantly done by men. For example, with 98.5% of the workers being male, carpentry was the fourth male-dominated occupation in the USA in 1999, and there were about 1.5 million positions in 2006 [8].

There is evidence that workplace exposure to wood dust may cause adverse health effects in exposed workers, such as dermatitis, allergic and non-allergic respiratory effects and cancer. Dermatitis is a common health effect associated with exposure to wood dust. Wood, usually as sawdust or splinters, may affect the skin by mechanical action or by chemical irritation and sensitization. Respiratory effects in workers exposed to wood dust may also be caused by irritation or sensitization. A hypersensitivity reaction leading to asthma has been reported as a result of exposure to commonly used woods, including western red cedar, oak, mahogany, and redwood. The asthmatic reaction is believed to be species-specific. Respiratory symptoms and lung function decline can be caused by irritation of the bronchial tree, too. The main population of workers who suffer from respiratory problems are those who work in secondary wood products manufacturing, although these problems have been documented in sawmill workers. The National Institute for Occupational Safety and Health (NIOSH) considers both hardwood and softwood dust to be potentially carcinogenic for humans. The three types of cancer associated with wood dust exposure are nasal and sinus cavity cancer, lung cancer, and Hodgkin’s disease. In addition, there is evidence that combined effect of wood dust and tobacco smoke increases the health risks in workers exposed to wood dust [7, 9-11].

In the present study we compared the prevalence of respiratory symptoms in the last 12 months and the values of the spirometric parameters, as well as its relation to duration of exposure between a group of never-smoking male workers exposed to natural hardwood dust and a group of unexposed male workers (office workers), matched by age and duration of employment at the actual workplace.

## Methods

### Study design and setting

A cross-sectional study was carried out in the Department of Cardiorespiratory Functional Diagnostics at the Institute for Occupational Health of R. Macedonia, Skopje - WHO Collaborating Center for Occupational Health and GA^2^LEN Collaborating Center in the period August – December 2014. Prevalence of respiratory symptoms in the last 12 months and mean values of spirometric parameters was compared between a group of never-smoking male workers exposed to hardwood dust and a group of never-smoking male office workers. All study subjects gave their informed consent before entering the study.

### Subjects

We examined 37 male workers aged 33 to 62 years, employed in a company for parquet manufacture with duration of employment between 7 to 26 years. They worked in two large closed working areas in two working shifts lasting 8 hours and their working tasks included measuring, cutting and shaping different types of natural hardwood, such as oak, beech, ash and elm. The process control provided keeping of the exposure at permissible levels. During the working process the employees were protected by working outfits, gloves, glasses and masks. All examined parquet manufacturers were never-smokers, i.e. non-smokers who have never smoked at all, or have never been daily smokers and have smoked less than 100 cigarettes in their lifetime [12, 13].

In addition, an equal group of never-smoking male office workers matched to construction workers by age and duration of employment at the actual workplace was studied as a control.

In either group there were no subjects with chronic respiratory disease diagnosed by a physician (i.e. asthma, COPD, bronchiectasis, etc.), neither subject treated with bronchodilators and/or corticosteroids. In either group also there were no subjects in whom spirometry was contraindicated [14, 15].

### Questionnaire

An interviewer-led questionnaire was completed by all study subjects. The questionnaire included questions on work history (e.g., chronological list of jobs; description of job activities at the actual workplace; type, extent and duration of exposure; and use of protective equipment), respiratory symptoms in the last 12 months and their relatedness to the workplace, chronic respiratory diseases diagnosed by a physician, family history of chronic bronchitis or asthma (taking into account the first-degree relatives), environmental exposure to tobacco smoke (ETS), accompanying disease, and medication use.

Respiratory symptoms in the last 12 months (cough, phlegm, dyspnea, wheezing, and chest tightness) were documented using the European Community for Coal and Steel questionnaire (ECCS-87), and the European Community Respiratory Health Survey (ECRHS) questionnaire [16, 17]. The work-relatedness of the respiratory symptoms was defined as more than usual cough, phlegm, dyspnea, wheezing, and chest tightness during daily work [18].

ETS or passive smoking or second-hand smoking was defined as an exposure to tobacco combustion products from smoking by others (at home, workplace, etc.), i.e. as a presence of at least one smoker in the household and/or at the workplace [19, 20].

### Baseline spirometry

The baseline spirometry, including measures of forced vital capacity (FVC), forced expiratory volume in one second (FEV_1_), FEV_1_/FVC ratio, and maximal expiratory flow at 25%, 50%, 75%, and 25-75% of FVC (MEF_25_, MEF_50_, MEF_75_, and MEF_25-75_, respectively), was performed in all subjects using spirometer Ganshorn SanoScope LF8 (Ganshorn Medizin Electronic GmbH, Germany) recording the best result from three measurements of the values of FEV_1_ which were within 5% of each other. The results of spirometry were expressed as percentages of the predicted values according to the actual recommendations of the European Respiratory Society (ERS) and the American Thoracic Society (ATS) [14, 15].

### Statistical analysis

Continuous variables were expressed as mean values with standard deviation (SD), and the nominal variables as numbers and percentages. Analyses of the data involved testing the differences in prevalence, comparison of the means, as well as testing the association between respiratory symptoms in the last 12 months and mean values of the MEF parameters (less or more than 60% of the predicted value) and duration of the exposure at the actual workplace (less or more than 15 years). Chi-square test (or Fisher’s exact test where appropriate) was used for testing difference in the prevalence. Comparison of spirometric measurements was performed by independent-samples *T-*test. A *P*-value less than 0.05 was considered as statistically significant. Statistical analysis was performed using the Statistical Package for the Social Sciences (SPSS) version 11.0 for Windows.

## Results

Demographic characteristics of the study participants are given in Table 1.

**Table 1:** Demographics of the study subjects

Variable	Parquet manufacturers (n = 37)	Office workers (n = 37)
Age (years)	44.9 ± 7.8	45.2 ± 5.4
BMI (kg/m^2^)	25.1 ± 3.1	25.8 ± 3.8
Duration of employment (actual workplace, years)	17.4 ± 4.3	18.1 ± 3.9
Duration of employment less than 15 years	21 (56.8%)	19 (51.3%)
Duration of employmentmore than 15 years	16 (43.2%)	18 (48.7%)
Family history of asthma/chronic bronchitis	5 (13.5%)	4 (10.8%)
Environmental ETS	18 (48.6%)	15 (40.5%)
Accompanying diseases		
Arterial hypertension	5 (13.5%)	5 (13.5%)
Diabetes mellitus type 2	2 (5.4%)	3 (8.1%)
Musculoskeletal disorders	6 (16.2%)	4 (10.8%)
Peptic ulcer	3 (8.1%)	4 (10.8%)

Numerical data are expressed as mean value with standard deviation; frequencies as number and percentage of study subjects with certain variable. BMI: body mass index; kg: kilogram; m: meter; ETS: exposure to tobacco smoke.

Prevalence of overall respiratory symptoms in the last 12 months was higher in parquet manufacturers than in office workers but the difference did not reach statistical significance.

**Figure 1:**
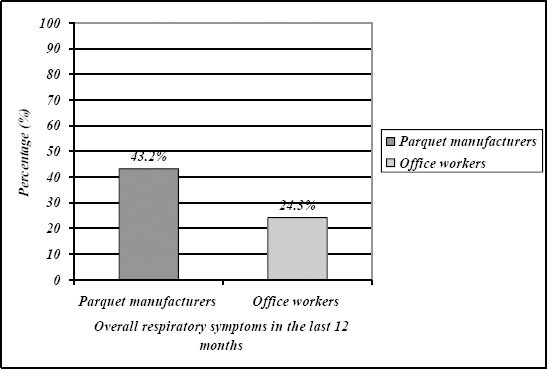
*Prevalence of overall respiratory symptoms in the last 12 months*.

**Figure 2:**
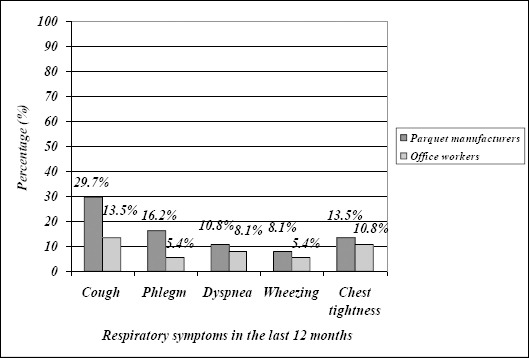
*Prevalence of particular respiratory symptoms in the last 12 months*.

Prevalence of particular respiratory symptoms was also higher in parquet manufacturers than in office workers with statistically significant difference for cough and phlegm (Table 2, Figure 1 and Figure 2).

**Table 2:** Prevalence of respiratory symptoms in the last 12 months

Respiratory symptoms in the last 12 months	Parquet manufacturers (n = 37)	Office workers (n = 37)	*P*-value*
Overall respiratory symptoms	16 (43.2%)	9 (24.3%)	0.092
Cough	11 (29.7%)	5 (13.5%)	0.044
Phlegm	6 (16.2%)	2 (5.4%)	0.021
Dyspnea	4 (10.8%)	3 (8.1%)	0.119
Wheezing	3 (8.1%)	2 (5.4%)	0.143
Chest tightness	5 (13.5%)	4 (10.8%)	0.138

Data are expressed as number and percentage of study subjects with certain variable.

* Tested by Chi-square test (or Fisher’s exact test where appropriate).

Majority of the respiratory symptoms in the last 12 months in parquet manufacturers were reported as work-related symptoms (13/16; 81.2%). The highest work-relatedness was reported for cough (8/11; 72.7%), phlegm (5/6; 83.3%) and dyspnea (3/4; 75.0%).

**Figure 3:**
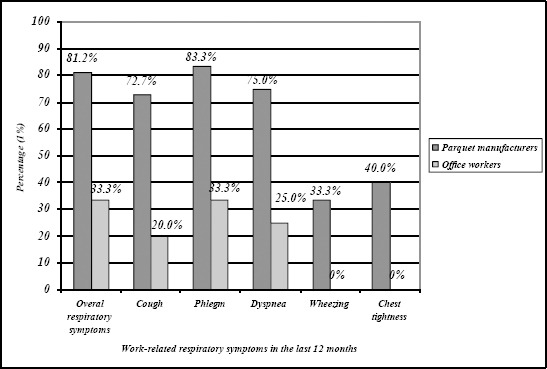
*Work-relatedness of the respiratory symptoms in the last 12 months*.

Work- relatedness of the respiratory symptoms was reported by three office workers with respiratory symptoms in the last 12 months (33.3%), i.e. by one office worker with cough, dyspnea and chest tightness (20.0%, 33.3% and 25.0%, respectively) (Figure 3).

Prevalence of overall respiratory symptoms in the last 12 months was significantly lower in the parquet manufacturers with duration of exposure at the actual workplace longer than 15 years than in the parquet manufacturers with duration of exposure at the actual workplace shorter than 15 years. In addition, significant difference was found in the prevalence of cough and phlegm in the parquet manufacturers with duration of exposure longer than 15 years as compared to those with shorter duration of exposure at the actual workplace (Table 3). Such difference in the office workers was not registered neither for overall respiratory symptoms in the last 12 months, nor for any particular respiratory symptom in the last 12 months.

**Table 3:** Prevalence of respiratory symptoms in the last 12 months in the parquet manufacturers with duration of exposure at the actual workplace more and less than 15 years

Respiratory symptoms in the last 12 months	Duration of exposure > 15 years	Duration of exposure < 15 years	*P*-value*
Overall respiratory symptoms	12/16 (75.0%)	4/16 (25.0%)	0.037
Cough	8/11 (72.7%)	3/11 (27.3%)	0.040
Phlegm	5/6 (83.3%)	1/6 (16.7%)	0.014
Dyspnea	2/4 (50.0%)	2/4 (50.0%)	1.000
Wheezing	2/3 (66.6%)	1/3 (33.3%)	0.083
Chest tightness	2/5 (40.0%)	3/5 (60.0%)	0.108

Data are expressed as number and percentage of study subjects with certain variable.

* Tested by Chi-square test (or Fisher’s exact test where appropriate).

The mean values of baseline spirometric parameters with exception of the mean FVC value were significantly lower in the parquet manufacturers than in the office workers (Table 4).

**Table 4:** Mean baseline values of spirometric parameters

Spirometric parameter	Parquet manufacturers	Office workers	*P*-value*
	(n = 37)	(n = 37)	
FVC (%pred)	92.3 ± 14.7	94.2 ± 11.4	0.088
FEV_1_ (%pred)	82.6 ± 10.2	86.8 ± 9.4	0.034
FEV_1_/FVC	0.75 ± 0.05	0.78 ± 0.03	0.037
MEF_25_ (%pred)	70.3 ± 8.9	76.9 ± 7.4	0.029
MEF_50_ (%pred)	64.6 ± 13.8	72.7 ± 10.5	0.011
MEF_75_ (%pred)	60.2 ± 16.7	69.7 ± 11.8	0
MEF_25-75_ (%pred)	65.9 ± 14.1	70.8 ± 12.9	0.006

Data are expressed as mean value with standard deviation. FVC: forced vital capacity; FEV_1_: forced expiratory volume in one second; MEF_75_, MEF_50_, MEF_25_, MEF_25-75_: maximal expiratory flow at 25, 50%, 75% and 25-75% of FVC, respectively; % pred: % of predicted value.

* Compared by Independent-samples *T-*test.

The mean values of all spirometric parameters were significantly lower in the parquet manufacturers with duration of exposure at the actual workplace longer than 15 years as compared to those with duration of exposure at the actual workplace shorter than 15 years (Table 5). Such difference in the office workers was registered only for MEF_75_ and MEF_25-75_ (71.8% *vs*. 67.4%; *P* = 0.028 and 72.6% *vs*. 68.1%; *P* = 0.037, respectively).

**Table 5:** Mean baseline values of spirometric parameters in the parquet manufacturers with duration of exposure at the actual workplace longer and shorter than 15 years

Spirometric parameter	Duration of exposure	Duration of exposure	*P*-value*
	> 15 years	< 15 years	
FVC (%pred)	90.4 ± 12.6	93.2 ± 10.9	0.038
FEV_1_ (%pred)	79.2 ± 9.8	84.3 ± 11.4	0.029
FEV_1_/FVC	0.74 ± 0.02	0.76 ± 0.04	0.022
MEF_25_ (%pred)	68.5 ± 11.7	72.6 ± 10.8	0.012
MEF_50_ (%pred)	61.3 ± 10.2	67.1 ± 11.9	0.002
MEF_75_ (%pred)	56.1 ± 13.4	64.2 ± 12.8	0
MEF_25-75_ (%pred)	62.7 ± 14.3	68.9 ± 12.1	0.003

Data are expressed as mean value with standard deviation. FVC: forced vital capacity; FEV_1_: forced expiratory volume in one second; MEF_75_, MEF_50_, MEF_25_, MEF_25-75_: maximal expiratory flow at 25, 50%, 75% and 25-75% of FVC, respectively; % pred: % of predicted value.

* Compared by Independent-samples *T-*test.

## Discussion

Respiratory effects of workplace exposure to wood dust are evaluated in a number of studies. These studies produced somewhat inconsistent results depending upon the type, level and duration of the workplace exposure to wood dust, the characteristics of the examined workers (age, smoking status, etc.) and the type of their work activities, as well as upon the type of the study.

In the present study we compared the prevalence of respiratory symptoms in the last 12 months and the mean values of spirometric parameters, as well as their relation to duration of the workplace exposure between the group of never-smoking male workers exposed to hardwood dust and the group of never-smoking male office workers matched by age and duration of employment at the actual workplace. Both groups had similar demographic characteristics regarding the body mass index value, family history of asthma/chronic bronchitis, environmental exposure to ETS and accompanying diseases.

We found higher prevalence of the respiratory symptoms in the last 12 months in the parquet manufacturers as compared to their prevalence in the office workers with statistically significant difference for cough and phlegm. Majority of the respiratory symptoms in the last 12 months in the parquet manufacturers were work-related and there was a close relationship between their prevalence and the duration of the workplace exposure to wood dust. Similar results were obtained in several studies which investigated the relationship between respiratory symptoms and workplace exposure to wood dust, such as the cross-sectional study performed by Okwari et al. including 221 workers exposed to wood dust in timber markets from Calabar, Nigeria [21], the cross-sectional study performed by Arbak et al. including 64 Turkish furniture-decoration students [22], the cross-sectional study performed by Sclhünssen et al. including 54 Danish furniture factory workers [23], and the cross-sectional study performed by Douwes et al. including 772 New Zealander pine sawmill workers [24]. Higher prevalence of respiratory symptoms in workers exposed to wood dust than in office workers was also reported by Ige & Onadeko [25] in a cross-sectional study including 500 sawmillers from Ibadan, Nigeria, but the prevalence of phlegm was twofold higher than its prevalence registered in the actual study (34.3% *vs*. 16.2%) that may be due to the specific wood types and specific work activities of these workers. In addition, in a cross-sectional study including workers employed in dusty occupations, Danilova et al. [26] reported similar prevalence of respiratory symptoms in furniture manufacturers in which the workplace exposure was somewhat different as compared to the workplace exposure of the wood workers examined in the actual study.

The mean values of the spirometric parameters in the parquet manufacturers were lower as compared to their mean values in the office workers. Statistically significant difference was registered for all measured spirometric parameters with exception of the mean values of FVC. Similar results were obtained in several studies which investigated the effect of different types of wood dust on the lung function of exposed workers. In a cross-sectional study including 50 males working in sawmills in different areas in Bhavnagar city, India, Kacha et al. registered significantly lower values of FVC, FEV_1_, MEF_50_, MEF_25_ and PEFR as compared to their predicted values [27]. In a cross-sectional study including 328 smoking and non-smoking woodworkers, Osman & Pala registered significantly lower mean values of FVC and FEV_1_ in both smokers and non-smokers as compared to their mean values in the controls [28]. In addition, in a cross-sectional study including 46 non-smoking Pakistani woodworkers, Meo registered significantly lower mean values of FVC and FEV_1_ as compared to their mean values in unexposed controls [29]. Dudhmal et al. in their study, including 30 Indian non-smoking sawmill workers, found significantly lower mean values of FEV_1_ and PEFR as compared to their predicted values, whereas regarding the mean FVC value such difference was not found [30]. In the actual study we found significant relation between the reduction of the mean values of the measured spirometric parameters and the duration of exposure to wood dust. Similar results were registered in the cross-sectional studies performed by Kacha et al. and Meo [27, 29] mentioned above. In a cross-sectional study including 145 South African non-smoking woodworkers and 152 matched controls, Shamssain reported significantly lower FEV1/FVC ratio in woodworkers than in the controls. Furthermore, the FEV1/FVC ratio was significantly lower in woodworkers with duration of employment longer than 10 years as compared to those exposed shorter than 10 years [31]. On the contrary, in a longitudinal study including 31 Italian non-smoking forestry workers with assessment of the lung function at least five times in the average period of 12 years, Inocenti et al. did not register significant difference in the VC and FEV_1_ loss between the woodworkers and the controls [32].

The present study had some limitations. First, relatively small number of subjects in the study groups could have certain implications on the data obtained and its interpretation. Second, environmental measurements were not performed, so we could not document the effect of the type and the level of workplace exposure in these workers. Third, as in the case of any cross-sectional study, the impact of healthy workers’ effect (HWE) on the data obtained could not be excluded. The strength of the study is the investigation of respiratory effects of specific occupational exposure in the wood-working environment independently from the effects of tobacco smoke.

In conclusion, in a cross-sectional study including never-smoking male parquet manufacturers we found higher prevalence of respiratory symptoms in the last 12 months and lower values of spirometric parameters than in matched office workers. There was a close relationship both between the prevalence of the respiratory symptoms in the last 12 months and the reduction of the spirometric parameters and the duration of the workplace exposure to wood dust. Our results suggest adverse respiratory effects of workplace exposure to hardwood dust indicating the need of reduction of harmful workplace exposure via proper engineering control, as well as the need of regular medical examinations in order to identify affected workers and thereby institute adequate measures.

## Authors Participations

DB participated in the study design, writing the protocol, data collection, managing the analyses of the study, and writing all versions of the manuscript. SP participated in the study design, writing the protocol, managing the analyses of the study, as well as writing all versions of the manuscript. JM performed the statistical analysis and participated in the managing of the analyses of the study. DB and JM participated in the data collection and in the managing of the analyses of the study. All authors read and approved the final manuscript.
